# Approximate Subject Specific Pseudo MRI from an Available MRI Dataset for MEG Source Imaging

**DOI:** 10.3389/fninf.2017.00050

**Published:** 2017-08-08

**Authors:** Bakul Gohel, Sanghyun Lim, Min-Young Kim, Hyukchan Kwon, Kiwoong Kim

**Affiliations:** Center for Biosignals, Korea Research Institute of Standards and Science Daejeon, South Korea

**Keywords:** MRI, pseudo MRI, ICP registration, MEG source imaging, sourcemodel, headmodel

## Abstract

Computation of headmodel and sourcemodel from the subject's MRI scan is an essential step for source localization of magnetoencephalography (MEG) (or EEG) sensor signals. In the absence of a real MRI scan, pseudo MRI (i.e., associated headmodel and sourcemodel) is often approximated from an available standard MRI template or pool of MRI scans considering the subject's digitized head surface. In the present study, we approximated two types of pseudo MRI (i.e., associated headmodel and sourcemodel) using an available pool of MRI scans with the focus on MEG source imaging. The first was the first rank pseudo MRI; that is, the MRI scan in the dataset having the lowest objective registration error (ORE) after being registered (rigid body transformation with isotropic scaling) to the subject's digitized head surface. The second was the averaged rank pseudo MRI that is generated by averaging of headmodels and sourcemodels from multiple MRI scans respectively, after being registered to the subject's digitized head surface. Subject level analysis showed that the mean upper bound of source location error for the approximated sourcemodel in reference to the real one was 10 ± 3 mm for the averaged rank pseudo MRI, which was significantly lower than the first rank pseudo MRI approach. Functional group source response in the brain to visual stimulation in the form of event-related power (ERP) at the time latency of peak amplitude showed noticeably identical source distribution for first rank pseudo MRI, averaged rank pseudo MRI, and real MRI. The source localization error for functional peak response was significantly lower for averaged rank pseudo MRI compared to first rank pseudo MRI. We conclude that it is feasible to use approximated pseudo MRI, particularly the averaged rank pseudo MRI, as a substitute for real MRI without losing the generality of the functional group source response.

## Introduction

Currently, magnetoencephalography (MEG) is one of the most important non-invasive neuroimaging modalities used to investigate various brain spatiotemporal neural dynamics and cognitive functions. Typically, MEG-based studies involve anatomical localization of the source from the recorded neural activity at MEG sensors. This process essentially requires co-registration of anatomical information of the brain from MRI scan and MEG sensors in a common three-dimensional space. Hence, participants of MEG experiments also require visiting an MRI recording facility, which is often located away from the MEG setup, thus requiring an additional experimental setting. Moreover, MRI scanning involves significant time and money resources, and participant drop out is often a costly inconvenience. One alternative approach to bypass MRI scanning is to generate approximated anatomical head or brain models, or pseudo MRI, from an available pool of MRI scans.

Anatomical source localization of sensor MEG (and EEG) activity requires the *a priori* construction of the headmodel and sourcemodel (Wendel et al., 2009). The headmodel represents the volume conductor model (for the electric current or magnetic field) for the source activity in the brain to the sensors. The sourcemodel represents the source locations in the brain from where the source activity is to be reconstructed. Anatomical information from the MRI scan of the subject is generally used to create the headmodel and sourcemodel. However, in the absence of an MRI scan of the subject, pseudo MRI (the headmodel and sourcemodel) can be approximated in one of two ways. Firstly, it can be done by warping surface landmarks of a standard MRI template (e.g., averaged MNI, ICMP-152, colin27, averaged from the target population MRI) to the digitized head landmarks/scalp surface of a subject (Fuchs et al., 2002; Darvas et al., 2006; Tadel et al., 2011). Or, researchers can select an individual MRI scan from an MRI dataset that is registered to a subject's digitized head surface with minimum objective registration error (ORE) (Holliday et al., 2003). Recently, the use of pseudo MRI in the absence of real MRIs has also been suggested; but with taking into consideration the source localization error in the absence of intracranial structure information (Gross et al., 2013). In a previous EEG-based study, pseudo MRI was computed using thin plate spline warping of the 10−20 electrode placement system, like grid landmarks on the head of the participant to corresponding landmarks on a standard template (Darvas et al., 2006). This study showed a mean source localization error magnitude of 15.2 ± 5.9 mm in the atlas space, while error magnitude exceeded 25 mm for peripheral brain regions for approximated pseudo MRI compared with real MRI information. Moreover, Darvas et al. (2006) also evaluated rigid body transformation with scaling (Fuchs et al., 2002) and observed a maximum source localization error exceeding 35 mm. This is likely because scaling was based only on three fiducial landmarks. Similarly, Brainstorm software provides rigid body transformation with scaling and warping transformation-based functionalities to approximate pseudo MRI from template MRI using information from the subject's digitized head surface (Tadel et al., 2011). In an EEG-based study, average approximated realistic MRI-based models, such as surface-based models or leadfields, were computed from a target population consisting of 305 MRIs (Valdés-Hernández et al., 2009). The resulting average approximated model was closer to all individuals of the target population in terms of shape and seemed to perform marginally better than the MNI-shaped model. However, the electrode position for an individual was directly projected from the template MRI, and the error matrix thus disregarded the electrode positioning error (Valdés-Hernández et al., 2009).

In a MEG-based study, instead of using single template MRIs, pseudo MRI was approximated by selecting individual MRIs from a dataset of a total of 27 MRIs that optimally registered to the subject's digitized head surface (Holliday et al., 2003). This study showed a mean source localization error of about 6 mm (using 27 subjects and only eight dipole locations in each subject) while in 90% of the cases the source localization error fell below 12.5 mm. Additionally, these researchers observed a functional group brain response (at the source level) similar for both real and pseudo MRI in response to a verbal fluency task (Holliday et al., 2003). However, this estimate was based on six participants only.

The studies mentioned above have mainly focused on the source localization error for the simulated source at the individual level. Typically, MEG-based brain functional mapping is carried out as a group study where the conclusion is drawn from the mean group effect. It is worth noting that there exists functional and anatomical variability across subjects (Xiong et al., 2000; Nieto-Castanon and Fedorenko, 2012), error from the registration of digitized head surface or fiducial to real MRI (Whalen et al., 2008; Chiarelli et al., 2015), error from registration of real MRI to template MRI or atlas (Hinds et al., 2009; Ghosh et al., 2010; Nieto-Castanon and Fedorenko, 2012), and error from head movement inside the Dewar during MEG signal recording. Despite the advantage of having real MRIs for each of subjects, the aforementioned functional and anatomical inter-individual discrepancies cause blurring of the brain response. Consequently, due to the large inter-subject variability in brain responses, a little information may be lost in a group effect after analyzing the data using the approximated pseudo MRI instead of real MRI (Holliday et al., 2003).

The goal of the present study was multifold. First, we aimed to find an approximated pseudo MRI from an MRI dataset that best matched a subject's digitized head surface in terms of head shape and size. The effective strength of MRI information further increased as we considered scaling these MRIs during our search for the best pseudo MRI. Second, in addition to using single best substitute pseudo MRI with the lowest ORE, we approximated a subject-specific averaged pseudo MRI (i.e., sourcemodel and headmodel) by averaging the first **n** number of pseudo MRIs that optimally registered to subject's digitized head surface. Given that the source localization error for different pseudo MRIs is likely to be in different orientations, averaging them might neutralize error to a certain extent. Third, we aimed to evaluate the similarity or difference in functional group source response for the real MRI and approximated pseudo MRI using real functional MEG signals.

## Materials and methods

### Dataset

Data used in the preparation of this work were obtained from the MGH-USC Human Connectome Project (HCP) database. The HCP project (Principal Investigators: Bruce Rosen, M.D., Ph.D., Martinos Center at Massachusetts General Hospital; Arthur W. Toga, Ph.D., University of California, Los Angeles, Van J. Weeden, MD, Martinos Center at Massachusetts General Hospital) is supported by the National Institute of Dental and Craniofacial Research (NIDCR), the National Institute of Mental Health (NIMH), and the National Institute of Neurological Disorders and Stroke (NINDS). Collectively, the HCP is the result of efforts of co-investigators from the University of California, Los Angeles, Martinos Center for Biomedical Imaging at Massachusetts General Hospital (MGH), Washington University, and the University of Minnesota (Larson-Prior et al., 2013).

From the database, we used pre-processed MRI and 3D digitized head surface data from 92 participants. The detailed experimental setup and data pre-processing pipeline are available at (http://www.humanconnectome.org/documentation/S500/HCP_S500+MEG2_Release_Reference_Manual.pdf). Briefly, structural MRI was recorded using a whole-head Magnes 3600 MRI scanner (4D Neuroimaging, San Diego, CA, USA) at a resolution of 0.7 mm that was subsequently changed to 1 mm. In addition to the structural MRI scan, 3D digitized head surface and head fiducial landmarks such as nasion, and the left and right pre-auricular landmarks of the subject were also provided. Head surface was digitized with more than 2,000 points. Moreover, the dataset also provided a precomputed cortical sheet sourcemodel with 8,004 source (dipole) locations registered to the common template (Conte69) space and precomputed single shell headmodel associated with each of the MRIs.

We also used pre-processed functional MEG signals from 82 participants performing a working memory task, available on the human connectome project web portal (Van Essen et al., 2013). Briefly, MEG data were recorded from participants performing the 0-back or 2-back working memory task in the visual sensory modality. We used data from only one out of two sessions, with eight blocks of the 0-back working memory task (sample to match task). In a single block of the 0-back working memory task, a target image of either a face or a tool was presented to a participant followed by a sequence of 10 images interspaced with a fixation cross. Each image and fixation cross was displayed for a duration of 2,000 and 500 ms, respectively. The participant had to press a button during the period of the subsequent presentation of the fixation cross whenever the image matched the target image. In total, there were 80 trials of the 0-back working memory task for each participant.

### Registration of digitized head surface and MRI

Estimation of source activity in the brain from MEG sensor activity requires registration of MEG sensors and brain anatomy in a common three-dimensional space. It is frequently achieved by registering head fiducial landmarks, and head surface digitized points to corresponding fiducial landmarks and scalp surface in an MRI image. For that, we adopted the following registration process. First, each MRI image was segmented into various tissue types (using *volumesegment function* with *scalpthreshold* value set to 0.08) using the Fieldtrip toolbox (Oostenveld et al., 2011). The MRI scalp surface in the form of mesh (with *50,000* vertices) was extracted from this segmented volume. Initially, digitized head surface and MRI scalp surface were registered to a common headspace (i.e., the BTi coordinate system) based on the nasion, and the right and left pre-auricular fiducial landmarks. Subsequently, a rigid body registration (six degrees of freedom, i.e., 3D rotation and 3D translation) was performed to fine-tune the co-registration between the digitized head surface and the MRI scalp surface using the point to plane iterative closet point algorithm (ICP) as provided in the Fieldtrip toolbox. Before this, we excluded the digitized head points that were more than 10 mm (i.e., < −10 mm in z-axis in BTi coordinate) below the plane formed by the nasion, left pre-auricular, and right pre-auricular fiducial landmarks. In practice, the head surface was often sampled with 50–150 digitized points. Thus, we used only 100 (uniformly distributed) out of more than 2,000 available head surface digitized points for the registration process.

The point to plane ICP algorithm registers source points to the target surface by minimizing objective error function that is the mean Euclidian distance between source points and target surface (Rusinkiewicz and Levoy, 2001; Park and Subbarao, 2003; Low, 2004). For the ICP registration process, various parameter values were set as follows: error “*Minimization”* parameter was set to “*Point to plane*,” “*Iteration*” number was set to *50*, “*WorstRejection”* was set to *0.05* (it rejects the given percentage of worst points having a higher magnitude of error), and “*Extrapolation”* was set to “*true”* (it leads to faster convergence by using gradient direction information). This ICP process minimizes the registration error between two surfaces, and returns the rigid body transformation matrix and residual ORE. Here, residual ORE represents the mean Euclidian distance between the digitized head surface points and the MRI scalp surface after registration; the lower the ORE the better the registration between two surfaces. However, the ICP algorithm is sensitive to local minima; the resultant solution may not necessarily be a perfect one. As the head size differs across subjects, it is important to incorporate a scaling parameter in the registration process. However, the rigid body ICP algorithm does not include a scaling parameter. Therefore, we adopted a serial approach in which ICP registration was performed after applying different levels of isotropic scaling to the MRI scalp surface. Indeed, the scaling factor can be estimated simultaneously with rotation and translation using another variant of ICP methods (Labadie et al., 2004). However, it also escalates the local minima due to the addition of more free parameters and may hence result in a higher registration error. Previous studies have also suggested better registration quality with the serial scaling approach (Whalen et al., 2008). In the present study, scaling was applied to the MRI scalp surface before the ICP registration process. We used scaling factors ranging between 0.85 and 1.15 with a resolution of 0.01 (~1 mm), which sufficiently covers all head sizes in the database. Moreover, we estimated the scaling parameters for the registration of the digitized head surface and real MRI scalp surface (with digitized head surface and MRI from the same subject), since MRI gradient inhomogeneity and the pre-processing (smoothing) of MRI images also introduce some level of scaling of the MRI image (Whalen et al., 2008).

### Pseudo MRI

**Figure 1:**
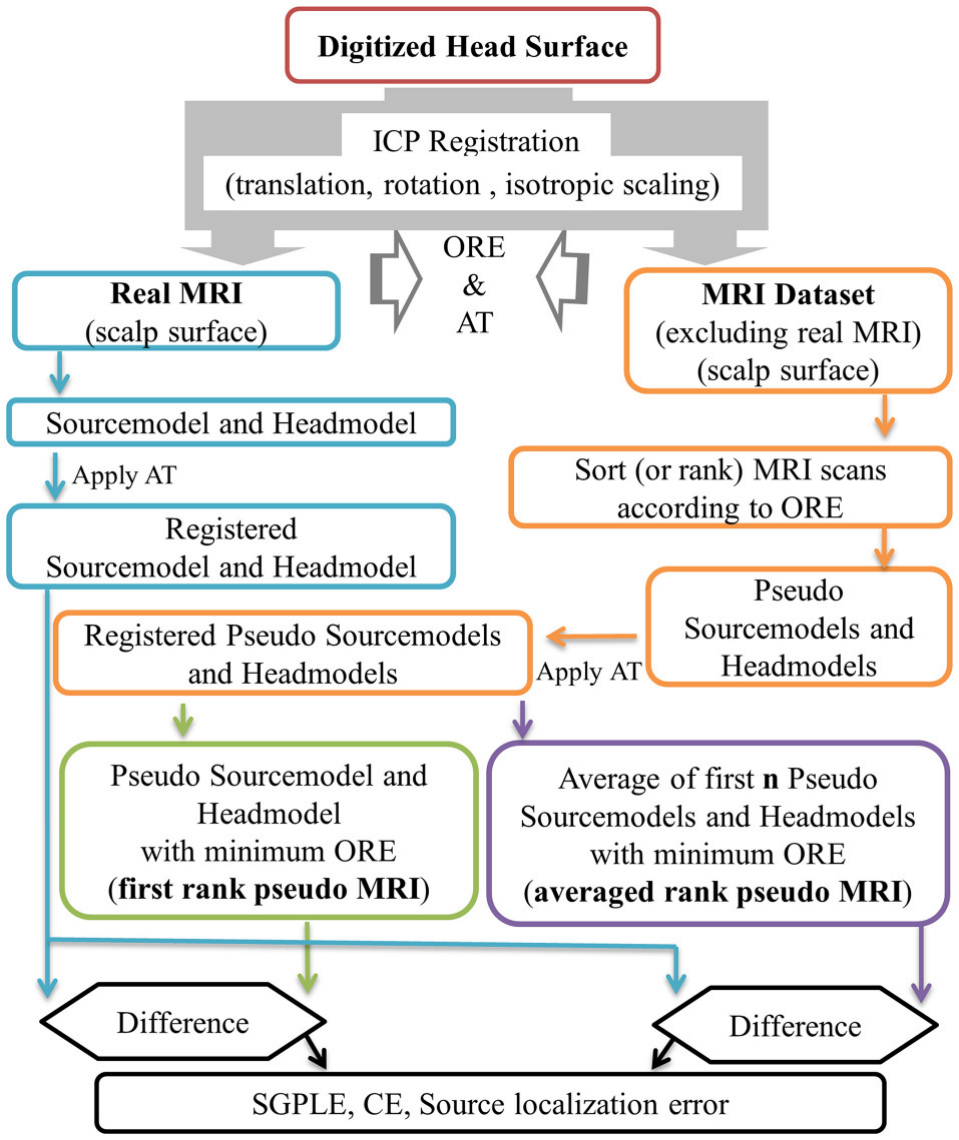
Outline of first rank pseudo MRI and averaged rank pseudo MRI approximation, and related error estimation. ORE, objective registration error; AT, affine transformation; SGPLE, source grid point location error; CE, Centroid error.

In the present study, approximate pseudo MRI essentially implies the approximation of headmodel and sourcemodel instead of the entire MRI image. An outline of pseudo MRI computation from the available pool of MRI images is provided in Figure 1. In the absence of an MRI scan from a given subject, we registered each MRI image (“*j*”) in the dataset (91 MRI scans that do not contain subject's own MRI scan) to the subject's digitized head surface (“*i*”). For this we used the aforementioned ICP registration process and estimated the rigid body transformation with scaling matrix (*T*^*i,j*^) and ORE^i, j^ (Equation 1). The estimated transformation (*T*^*i,j*^) was applied to the MRI associated precomputed sourcemodel and headmodel, resulting in a registered pseudo MRI, i.e., pseudo sourcemodel (*PSM*^*i,j*^, *i* ≠ *j*) and pseudo headmodel (*PHM*^*i,j*^, *i* ≠ *j*) and a real MRI, i.e., real sourcemodel (*RSM*^*i,j*^, *i* = *j*) and real headmodel (*RHM*^*i,j*^, *i* = *j*) for a given subject (Equations 2 and 3). Subsequently, all pseudo MRIs (*PSM*^*i,j*^ and *PHM*^*i,j*^) were ranked according to the ORE-value in ascending order for a given subject (*PSM*^*i,rank*^ and *PHM*^*i,rank*^, Equation 4). In the present study, we considered two kinds of pseudo MRIs. The first rank pseudo MRI [*PSM*(*FR*), *PHM*(*FR*), Equation 5] was the pseudo MRI with the lowest ORE. The averaged rank pseudo MRI [*PSM*(*AR*,**n**), *PHM*(*AR*,**n**, Equation 6] was the average of first **n** rank pseudo MRIs [*PSM*^*i,rank*=1 *to***n**^ and *PHM*^*i,rank*=1 *to***n**^, models are in the head coordinate system (BTi coordinate) after the ICP registration process] for a given subject. An averaged sourcemodel was directly computed by taking the centroid of the particular source grid point location (*r*) of first **n** rank pseudo sourcemodels (Equation 6a). This was not the case for the headmodel of the averaged rank pseudo MRI. Therefore, 3D brain volume was computed from the headmodel mesh for each of **n** number of pseudo headmodels and then averaged. This averaged 3D brain volume was thresholded with a 0.5 voxel value (volume overlap for more than 50% of the cases). An averaged headmodel mesh was recomputed using this thresholded averaged 3D brain volume (Equation 6b).

(1) $$\begin{array}{l} T^{i,j},\;ORE^{i,j}=register(Digitized\;head\;surface^{i},MRI\\ \;\;\;\;scalp\;surface^{j})\; \end{array}$$

(2a) $$PSM^{i,j}=T^{ij}\cdot MRI^{j}\left(sourcemodel\right),i\neq j$$

(2b) $$RSM^{i}=T^{ij}\cdot MRI^{j}\left(sourcemodel\right),i=j$$

(3a) $$PHM^{i,j}=T^{ij}\cdot MRI^{j}(headmodel),i\neq j$$

(3b) $$RHM^{i}=T^{ij}\cdot MRI^{j}(headmodel),i=j$$

(4a) $$\begin{array}{l} \{PSM^{i,\;rank}\;|\;rank=1,2,\ldots \;91\}=Sort_{as\;per\;ORE\;}\{PSM^{i,\;j}|\;j\\ \;=\;1,2,\ldots \;91\} \end{array}$$

(4b) $$\begin{array}{l} \{PHM^{i,\;rank}\;|\;rank=1,2,\ldots \;91\}=Sort_{as\;per\;ORE\;}\{PHM^{i,\;j}|\;j\\ \;=\;1,2,\ldots \;91\} \end{array}$$

(5a) $$PSM^{i}(FR)=PSM^{i,\;rank=1}$$

(5b) $$PHM^{i}(FR)=PHM^{i,\;rank=1}$$

(6a) $$PSM_{r}^{i}(AR,\;n)=\frac{\sum_{rank=1}^{n}PSM_{r}^{i,rank}\;(x,y,z)}{n}$$

(6b) $$PHM^{i}(AR,\;n)\text{​}=\text{​}mesh\left(\frac{\sum_{rank=1}^{n}volume(PHM^{i,\;rank})}{n}\text{​}>\text{​}0.5\text{​}\right)$$

Where “*i*” *and* “*j*” are the subject and associated MRI image index respectively, *T*, rigid body transformation with scaling matrix, *ORE*, objective registration error, *PSM*, pseudo sourcemodel, *PHM*, pseudo headmodel, *RSM*, real sourcemodel, *RHM*, real headmodel, *r*, source grid point location in sourcemodel, (*x,y,z*): *r*th location coordinates in head coordinate (after ICP registration), *FR*, first rank, *AR*, averaged rank.

### Event-related power (ERP) at source level

The event related field at sensor level was computed by averaging all trials within a subject. The sensor level averaged event-related field response was then projected to the source level using unconstrained minimum-norm estimate inverse solution as implemented in the Fieldtrip toolbox (Oostenveld et al., 2011). For computation of the inverse solution, the noise covariance matrix was determined from the baseline period (−1 to −0.5 s), leadfields were pre-whitened, the signal to noise ratio (SNR) value was set to three, and the normalization parameter value was set to 0.8. For each source grid point location in the sourcemodel, three orthogonally-oriented dipoles bring about three time series of source activities per source location. Thus, a single source activity time series [i.e., event-related power (ERP)] was computed by taking the norm of all three-source activity time series. The source ERP time series was normalized by subtracting the mean baseline amplitude and then dividing by the baseline standard deviation. For comparison, the source ERP time series for each of the sourcemodel grid point locations were computed for real MRI, first rank pseudo MRI, and averaged rank pseudo MRI.

### Source error computation

We evaluated two kinds of errors, subject level error and group level error. In the evaluation of the subject level error, we measured the Euclidian distance between the corresponding source grid point locations of the sourcemodel from real MRI and pseudo MRI for each subject. Since the source reconstruction errors are less model sensitive for MEG imaging modality, the source localization error mainly results from the deviation of grid point locations in the pseudo sourcemodel ($PSM_{r}^{i}$) from real sourcemodels ($RSM_{r}^{i}$). Therefore, the Euclidian distance between the corresponding grid point locations in the real sourcemodel and pseudo sourcemodel [i.e., the sourcemodel grid point location error (SGPLE)] directly provides an approximation of the possible source localization error (Equation 7).

(7) $$\begin{array}{r} SGPLE_{r}^{i}=\;distance\left(RSM_{r}^{i}\left(x,y,z\right),PSM_{r}^{i}\left(x,y,z\right)\right) \end{array}$$

Where $RSM_{r}^{i}\left(x,y,z\right)$ and $PSM_{r}^{i}\;\left(x,y,z\right)$ are the coordinate values for *i*th subject at *r*th source grid point location in the real sourcemodel and pseudo sourcemodel, respectively.

The source peak location, source strength, and source size (spatial distribution) are often the parameters of interest in functional brain mapping using MEG source imaging in group studies. Since the SGPLE for a given source location is likely to be different in terms of magnitude and orientation across subjects, it mainly causes blurring of the source, but it likely causes a lesser impact on the source's peak location in overall group effects. SGPLE in a group effect [henceforth called centroid error (*CE*)] provides a hint regarding the peak source localization error in a group response. In order to compute *CE* (Equation 8), we first randomly selected a number (group size) of subjects from our sample of 92. For each of the subjects in a group, we computed a vector that corresponded to the difference in the coordinates of the corresponding source grid point location between the real sourcemodel and pseudo sourcemodel. In other words, this vector represented the magnitude and orientation of SGPLE. Thereafter, these vectors from the subjects in a group were summed up and the computed norm of the resulting vector corresponded to the *CE* (Equation 8). We repeated this process 100 times (iterations) for each of the different group sizes range from 1 to 92. In fact, the peak location of the functional group source response depends additionally on the functional inter-subject variability. However, the *CE*, as computed here without taking account of the functional inter-subject variability, does not provide complete information but provides at least a hint toward a possible peak source localization difference between pseudo and real MRIs.

(8) $$\begin{array}{r} CE_{r}^{group\;size}=norm\left(\sum_{i=1}^{group\;size}\left(RSM_{r}^{i}\left(x,y,z\right)-PSM_{r}^{i}\;\left(x,y,z\right)\right)\right) \end{array}$$

Where $RSM_{r}^{i}\left(x,y,z\right)$ and $PSM_{r}^{i}\;\left(x,y,z\right)$ are the coordinate values for *i*th subject at *r*th source grid point location in the real and pseudo sourcemodel, respectively.

All the SGPLEs mentioned above for pseudo MRI were computed with reference to real MRI. In the current practical scenario, perfect registration of digitized head surface and MRI scalp surface is not guaranteed. Therefore, there also exists source localization error that arises from poor registration even when using real MRI. Furthermore, there exists inter-subject functional variability in source activity and brain anatomy registration error in matching MRI to a standard template (Xiong et al., 2000; Nieto-Castanon and Fedorenko, 2012). Altogether, these cause blurring of the functional source response even when using real MRI. Therefore, we performed a direct comparison of functional group source ERP response from both real and pseudo MRI. In practice, it is common to have 10–30 participants per group in MEG group studies. Therefore, we made a comparison of functional group source ERP responses from real and pseudo MRIs with 20 participants per group. We performed such comparisons 100 times (group iterations) in which 20 subjects were chosen randomly out of 82 participants in a single iteration. For functional source activity comparison, our focus was on observing similarity or difference in peak source strength, peak source location, and spatial distribution of source activity for real and approximated pseudo MRIs.

For the statistical evaluation of the peak source strength (for each group iteration), we first identified a peak source location with the highest source ERP magnitude (at time latency of around 0.108 s) in each of the left and right visual cortices, and for both real and pseudo MRIs. We used the bootstrapping approach to generate a null distribution for statistical evaluation of the difference in peak source strength. For each subject, baseline source ERP response at the peak source location was permuted (in the circular shift) homologously for both real and pseudo MRI, and then respectively, averaged across the subjects from one group (group size = 20) to generate the baseline group source ERP response. After that, we computed the difference in source magnitude between these baseline group source ERP responses from real and pseudo MRI, at each of the baseline time points. We repeated this process 100 times and combined all values to generate a null distribution (having 100 × 508.675 [sampling rate] × 0.5 s [baseline period] examples). Significant threshold values were determined by taking 0.5 and 99.5 quantile (*p* < 0.01) values from the null distribution. These threshold values were used to evaluate the significance of the difference in functional peak source strength (observed at a latency of around 0.108 s) between real and pseudo MRI.

## Results

### Sourcemodel grid point location error (SGPLE)

**Figure 2:**
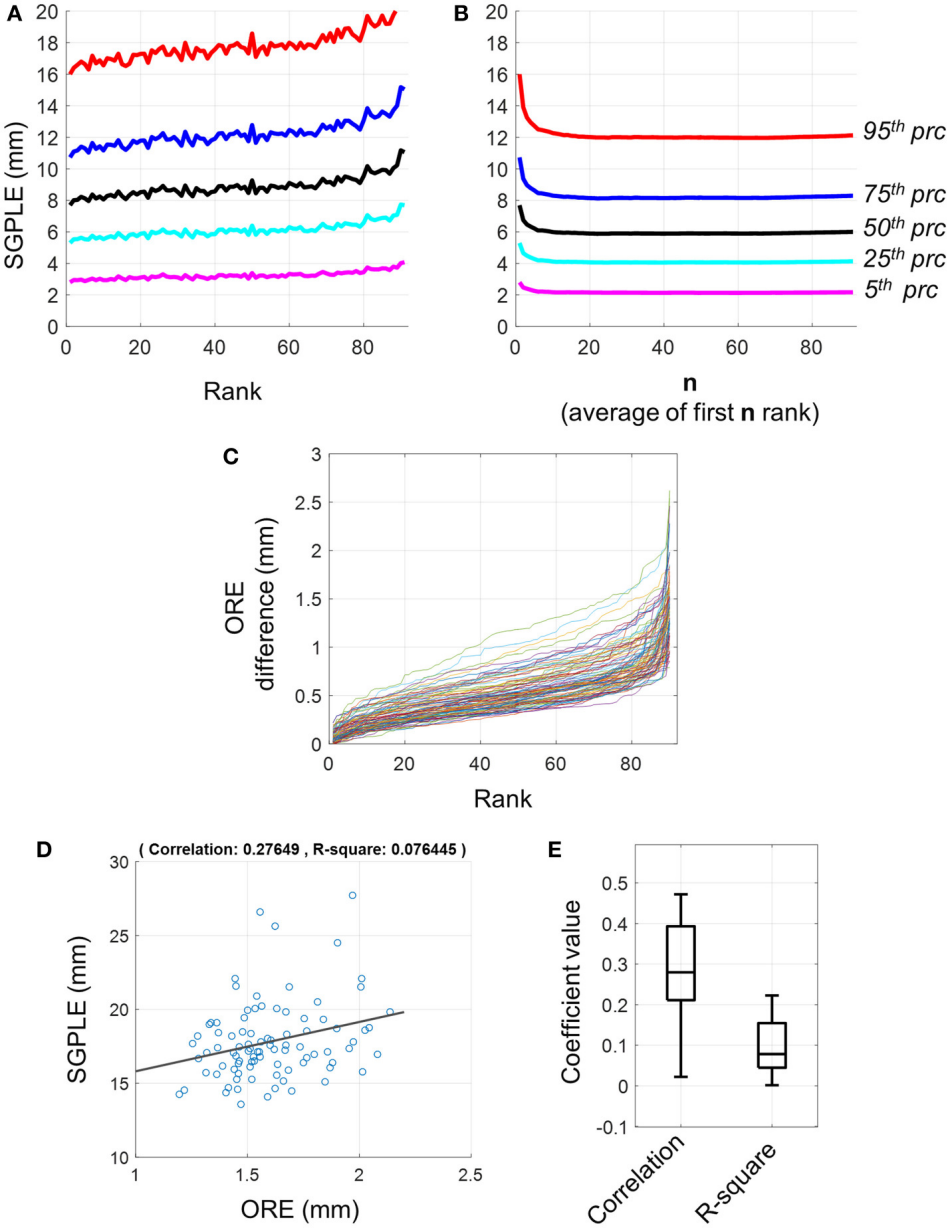
Sourcemodel grid point location error (SGPLE) and objective registration error (ORE). **(A)** The *meanSGPLE*_*prc*_ (Equation 7) for different rank of pseudo MRIs. **(B)** The *meanSLE*_*prc*_ (Equation 7) for averaged rank pseudo MRIs. **(C)** Difference in ORE between first rank pseudo MRI and 2nd to 91st rank pseudo MRIs (x-axis). Each line represents the result for one subject. There were total 92 subjects. **(D)** Scatter plot with the linear regression line for SGPLE against ORE for each of 91 pseudo MRI (dots) for one representative subject. **(E)** Distribution of the correlation coefficient and linear regression R-square coefficient for association between ORE and SGPLE across the subjects.

The *meanSGPLE*_*prc*_ values, that is *prc* = 5, 15, 25, 75, and 95 percentile values of SGPLE across source locations within a subject were extracted and then averaged, respectively across subjects (Equation 9). First rank pseudo MRI showed a mean upper bound SGPLE (i.e., *meanSGPLE*_95_) of about 16 mm (Figure 2A). However, SGPLE increased with the rank of pseudo MRI so that the difference in ORE was about 4 mm between first and last rank pseudo MRI (Figure 2A). The difference in ORE between two consecutive ranks of pseudo MRI was minimal (Figure 2C). Indeed, the difference in ORE between the first and second pseudo MRI was below 0.3 mm, while it was below 0.5 mm between the first and the 20th rank pseudo MRI for the majority of subjects. Moreover, there were weak to moderate associations between the ORE and SGPLE across subjects with correlation coefficients and R-squared regression coefficient ranging from ~0 to 0.48 and ~ 0 to 0.22, respectively (Figures 2D,E). These results indicate that a minimum rank pseudo MRI that has a minimum ORE does not necessarily imply minimum SGPLE.

(9) $$\begin{array}{r} meanSGPLE_{prc}=\frac{\sum_{i=1}^{N}\;prc\;of\;\left\{SGPLE_{r}^{i}\;|\;r=1,2,\ldots ,\;R\right\}}{N} \end{array}$$

Where *R* (= *8004*) is the number of source locations, *prc* is the percentile value, *N* (= 92) is the number of subjects.

In contrast to the first rank pseudo MRI, the upper bound of SGPLE (i.e., *meanSGPLE*_95_) for averaged rank pseudo MRI decreased from 16 to 12 mm after averaging 20 or more pseudo MRIs (***n*** ≥ 20; Figure 2B). This indicates that SGPLEs for different rank pseudo MRIs were in a different orientation and, consequently partially neutralized following the averaging of multiple pseudo MRIs. Since, SGPLE was optimal for averaging of 20 pseudo MRIs, averaged ranked pseudo MRI i.e., *PSM^i^*(*AR*, **n** = 20), was used for subsequent analysis.

**Figure 3:**
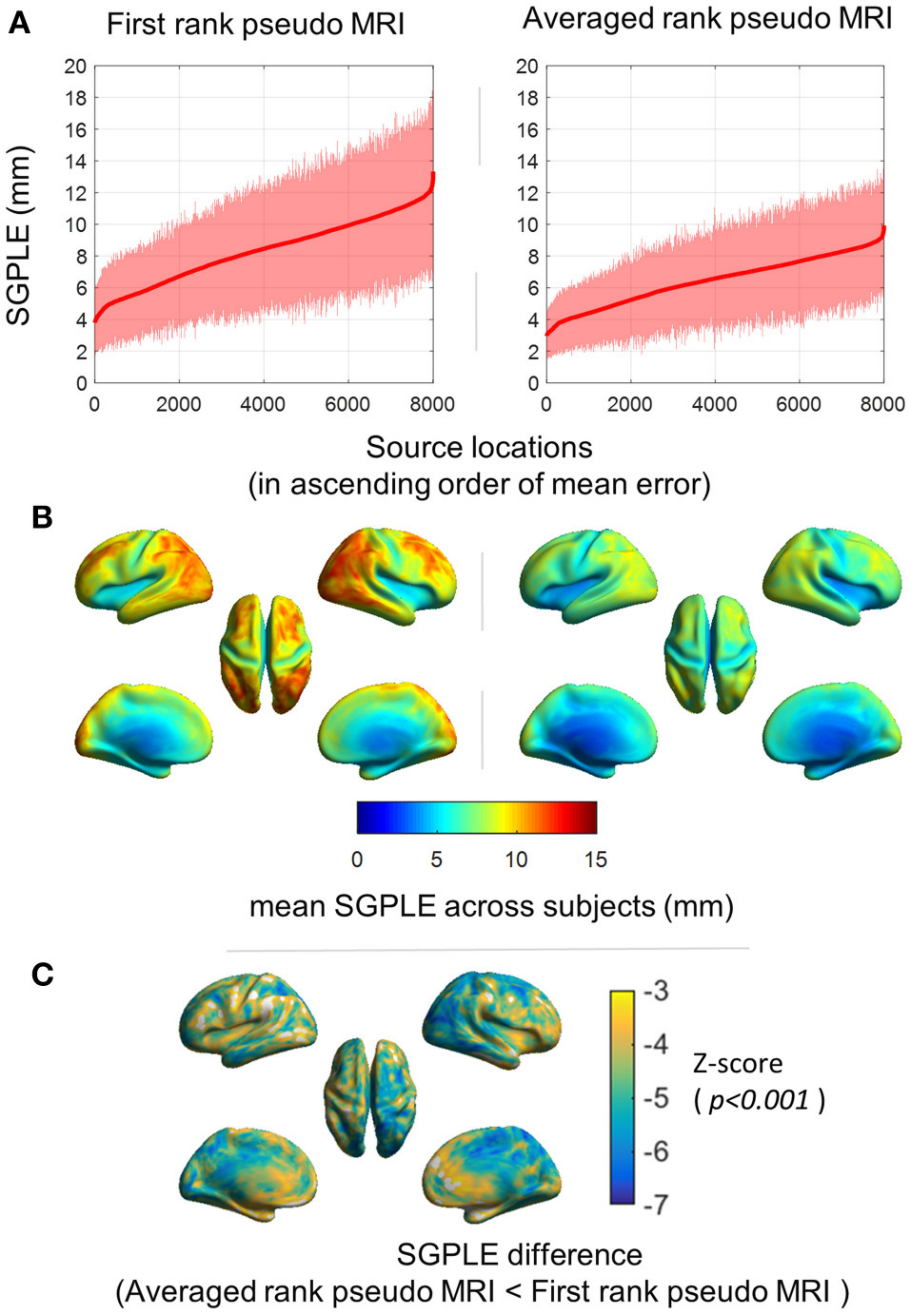
Spatial distribution of the sourcemodel grid point location error (SGPLE). **(A)** SGPLE for each of sourcemodel grid point locations across the subjects (mean ± standard deviation) where locations are ordered according to mean SGPLE. **(B)** Spatial distribution of mean of SGPLE across the subjects. **(C)** Spatial distribution of SGPLE difference (*p* < 0.001, *Wilcoxon signed-rank test, n* = 92 *subjects*) between first rank pseudo MRI and averaged rank pseudo MRI.

Spatial distribution of SGPLE for first rank pseudo MRI [i.e., $\;PSM_{r}^{i}\left(FR\right)$] and averaged rank pseudo MRI (i.e., $PSM_{r}^{i}$; *AR*, **n** = 20) is depicted in Figures 3A,B. The maximum SGPLE among source grid point locations was about 13 ± 5 mm (mean ± s.d. across subjects) and 10 ± 3 mm (mean ± s.d. across subjects) for first rank pseudo MRI and averaged rank pseudo MRI, respectively. In the case of spatial distribution, the peripheral brain regions, particularly the occipital, parietal, and lateral frontal cortices, showed more SGPLE compared with central brain regions. The majority of the source grid point locations showed significantly (*p* < 0.001, *Wilcoxon signed-rank test, n* = 92 *subjects*) lower SGPLE for averaged rank pseudo MRI compared with first rank pseudo MRI (Figure 3C).

### Centroid error (CE)

**Figure 4:**
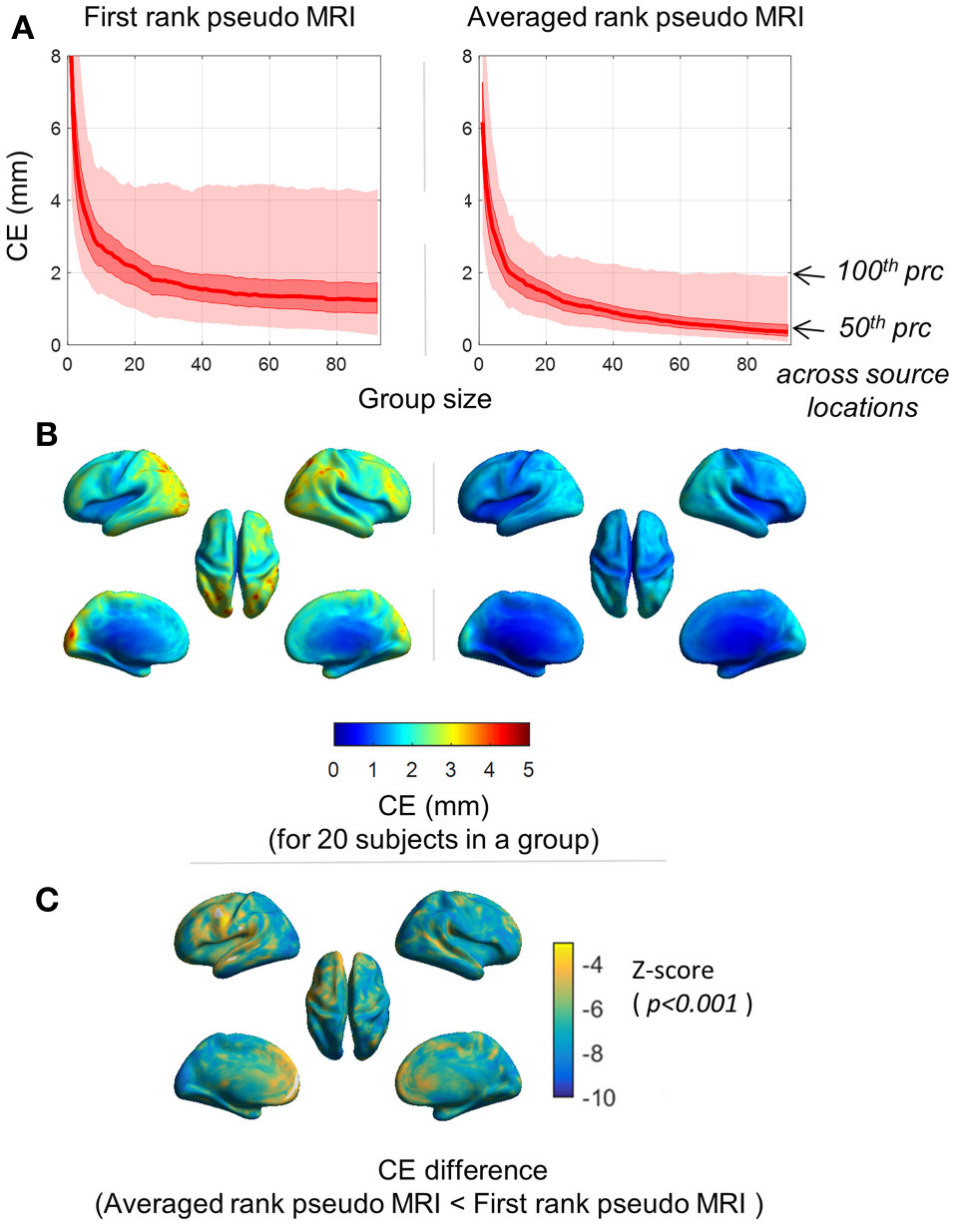
Spatial distribution of the centroid error (*CE*). *CE* represents the SGPLE in a group effect. **(A)** Distribution of mean *CE* (averaged over 100 iterations) across the sourcemodel grid point locations, for different group sizes. **(B)** Spatial distribution of mean *CE* (averaged over 100 iterations) for group size of 20 subjects. **(C)** Spatial distribution of *CE* difference (*p* < 0.001, *Wilcoxon signed-rank test, n* = 100 *iterations*) between first rank pseudo MRI and averaged rank pseudo MRI.

As expected, the magnitude of mean *CE* (averaged over 100 iterations) decreased with the increase in the number of subjects in a group (Figure 4A). With a group size exceeding 20 subjects, the maximum mean *CE* (100 percentile) across the source grid point locations was below 4.5 and 2.5 mm for the first rank pseudo MRI and average rank pseudo MRI, respectively (Figure 4A). The magnitude of mean *CE* (averaged over 100 iterations) was higher at the occipital-parietal and lateral-frontal cortices compared with central brain regions (Figure 4B). For a group size of 20 subjects, the majority of the source grid point locations showed significantly (*p* < 0.001, *Wilcoxon signed-rank test, n* = 100 *iterations*) lower *CE* for averaged rank pseudo MRI compared to first rank pseudo MRI (Figure 4C). These results indicate that source localization error for the peak source activation in a group effect is likely to be minimal and tolerable.

### Functional source localization error for group effect

**Figure 5:**
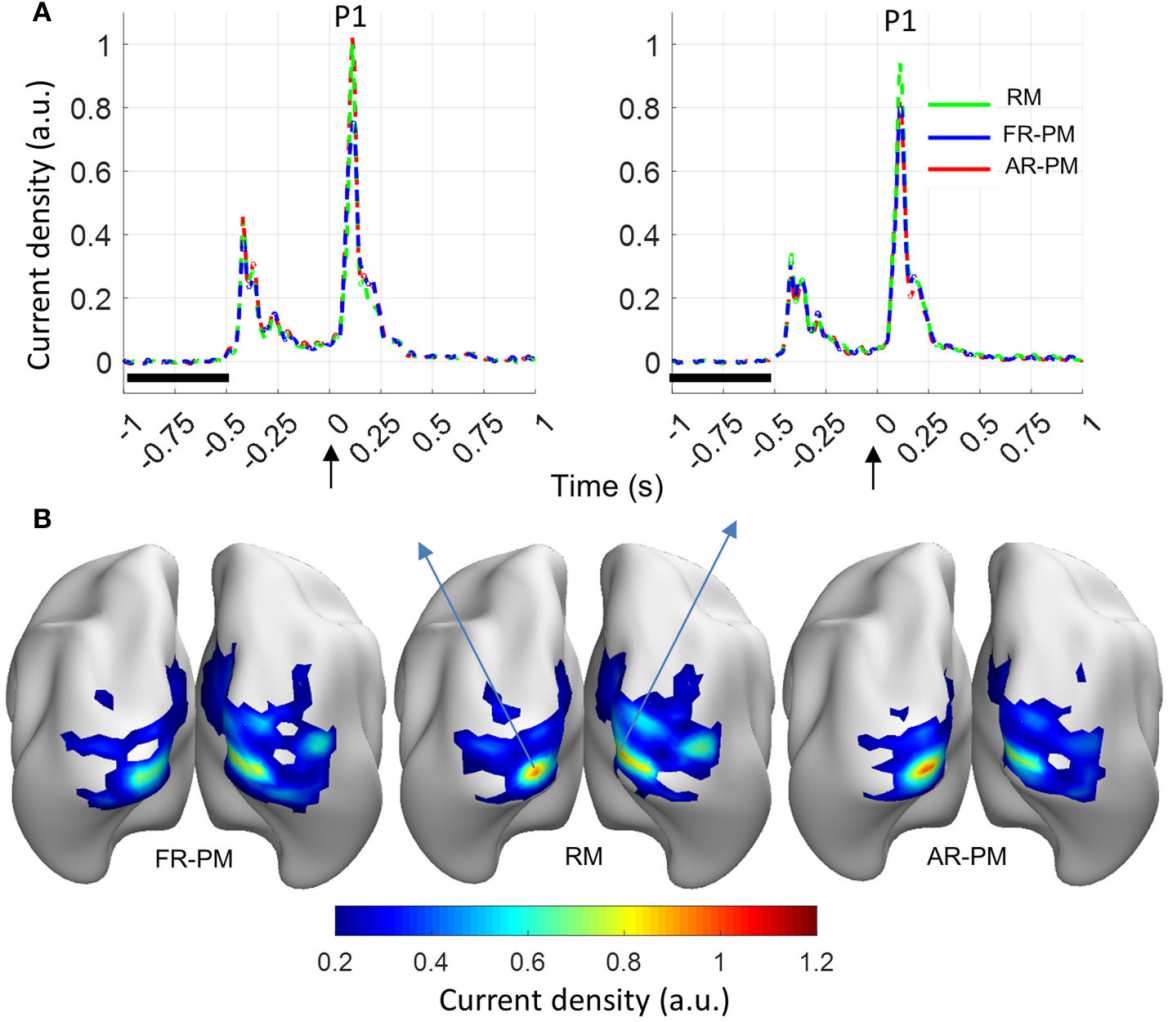
Functional group source response (event-related power [ERP]) from visual stimulation (averaged over 100 group iterations for 20 subjects in a group). **(A)** ERP time series for source locations with peak source activity at latency of 0.108 s (P1 peak) in left and right visual cortices. Baseline period: −1 to −0.5 s, Stimulus onset: 0 s. **(B)** Current density distribution for ERP peak (P1 component). a.u. arbitrary unit where a.u. = 1 corresponds to maximum source ERP value over the cortex for the real MRI. RM, real MRI; FR-PM, First rank pseudo MRI; and AR-PM, averaged rank pseudo MRI.

Functional group source ERP response (averaged across the 100 group iterations) showed a peak response (P1 component) at a time latency of 0.108 s and localized in the left and right primary visual cortices (Figure 5). Another peak response was observed at time latency −0.39 s, which was the stimulus off response from previous trials (Figure 5A). Noticeably, the source distribution of the ERP map at P1 latency (0.108 s) showed broadly similar source activity distribution for real MRI, first rank pseudo MRI, and averaged rank pseudo MRI (*n* = 20; Figure 5).

**Figure 6:**
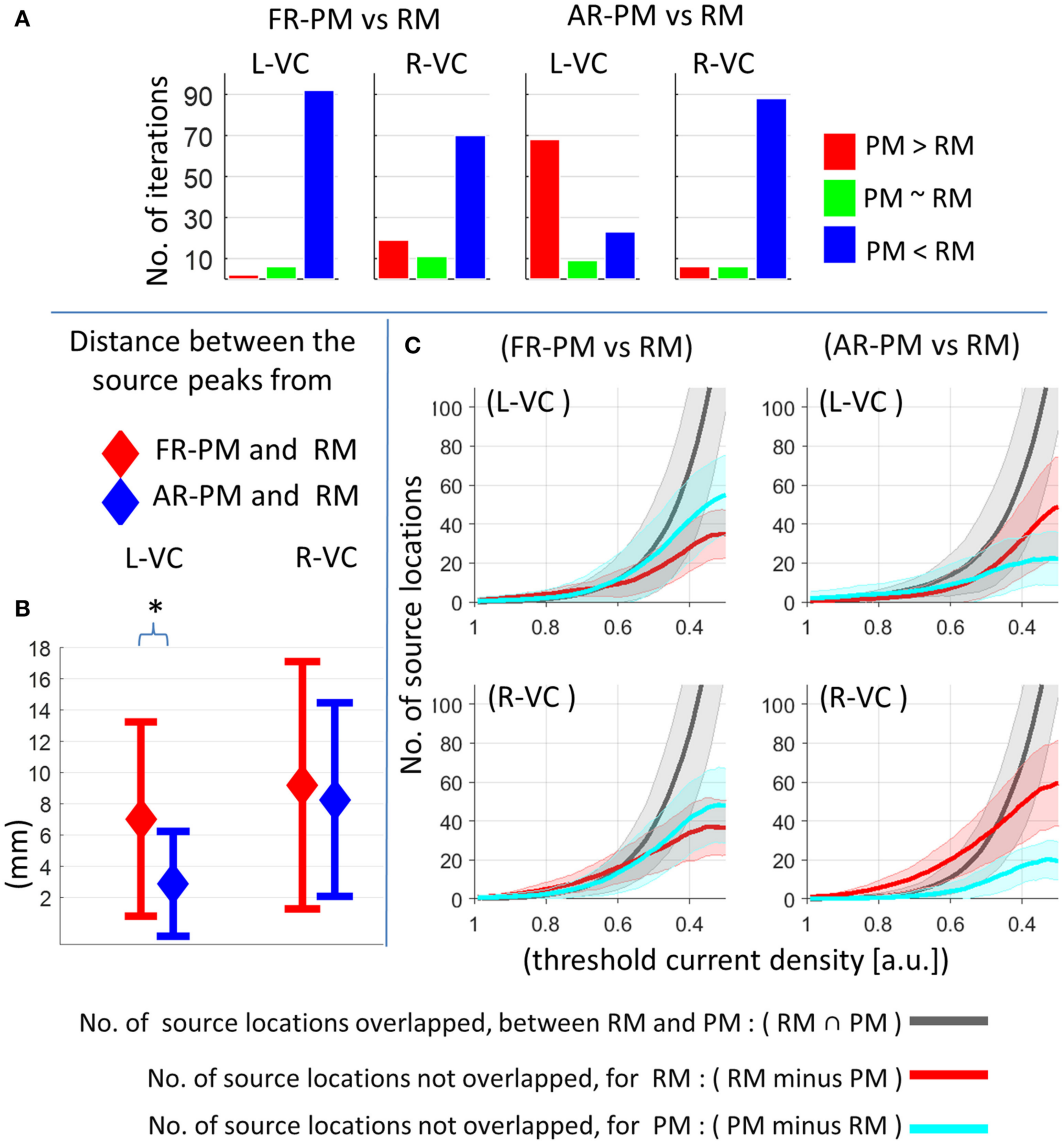
Comparison of functional group source ERP response between pseudo MRI and real MRI for 20 subjects in a group and 100 group iterations. **(A)** Comparison of peak source ERP magnitude for different group iterations. Y-axis represents number of group iterations showed significant difference for peak source ERP magnitude (*p* < 0.01: blue and red bar, *p* > 0.01: green bar, *bootstrap approach*). **(B)** Peak source localization error across the group iterations (mean ± standard deviation). *p < 0.01, *Wilcoxon signed-rank test, n* = 100 *group iterations*. **(C)** Overlap of activation region (no. of grid point locations) between real MRI and pseudo MRI after applying different levels of threshold value across the group iterations (mean ± standard deviation). RM, real MRI; PM, pseudo MRI; FR-PM, first rank pseudo MRI; AR-PM, averaged rank pseudo MRI; L-VC, left visual cortex; R-VC, right visual cortex; a.u., arbitrary unit where a.u. = 1 corresponds to maximum source ERP value for a given group iteration in visual cortex for real MRI.

We performed a statistical comparison of peak activation magnitude between pseudo and real MRIs for each of the group iterations [see Section Event-Related Power (ERP) at Source Level], and results are depicted in Figure 6A. There was significantly (*p* < 0.01) higher peak (P1) source strength for real MRI compared to first rank MRI in most (>70 out of 100) group iterations (Figure 6A). There were significantly (*p* < 0.01) higher peak magnitudes for averaged rank pseudo MRI compared with real MRI in most group iterations (68 out of 100) in the left visual cortex (Figure 6A). In contrast, there were significantly (*p* < 0.01) higher peak magnitudes for real MRI compared with averaged rank pseudo MRI in most group iterations (88 out of 100) in the right visual cortex (Figure 6A).

The peak source localization error (that is, the distance between peak source activity locations) in pseudo MRI and real MRI (at a latency of around 0.108 s) is depicted in Figure 6B. In the left visual cortex, peak source localization error was about 7.0 ± 6.2 mm (mean ± s.d. across group iterations) for first rank pseudo MRI and 2.8 ± 3.3 mm for averaged rank pseudo MRI, which was significantly (*p* < 0.001, *Wilcoxon signed-rank test, n* = 100 *group iterations*) lower than that of the first rank pseudo MRI. In the right visual cortex, the peak source localization error was about 9.1 ± 7.9 mm for first rank pseudo MRI and 8.2 ± 6.1 mm for averaged rank pseudo MRI. However, the overall peak source localization error decreased further with the increase in the number of subjects in a group (Supplementary information, Figures S1, S2).

The overlap of activated brain regions (number of sourcemodel grid point locations) between pseudo and real MRIs, after applying different levels of the threshold value to functional group source activity distribution at P1 peak (at a latency of around 0.108 s), are depicted in Figure 6C. For higher threshold values, there was an absent or minimal overlap of activated brain regions between real and pseudo MRI (Figure 6). Obviously, this was directly influenced by the peak source localization error. However, the overlap of activated brain regions [about >50% overlap for threshold value <0.4 (a.u: arbitrary units)] increased with the decrease of the threshold value (Figure 6C).

## Discussion

In the current study, we approximated pseudo MRIs (i.e., headmodel and sourcemodel) from an available MRI dataset using structural information of the subject's digitized head surface. We approximated source localization error at an individual and group level for pseudo MRI in reference to the subject's real MRI.

We observed larger SGPLE and *CE* mainly in the periphery rather than the center of the brain (Figures 2, 3). This is in line with a previous study reporting the similar spatial distribution of source localization error for approximated pseudo MRI (Darvas et al., 2006). The magnitude of the error that arose from brain surface-based registration of individual brain anatomy to a template brain (Hinds et al., 2009; Ghosh et al., 2010) was much lower than the observed SGPLE in the present study. A higher magnitude of SGPLE was found in the peripheral than the central region of the brain, and given the semi-spherical shape of the head SGPLE likely resulted from an error in the registration of digitized head surface to MRI scalp surface; a rotation rather than a translation error. Another likely contributing factor is the cortical gyrus folding pattern variability across subjects. As the head size differences between the subjects were already compensated through scaling during registration, the difference in brain size and shape in relative to head size and shape between the subjects was another contributing factor to the observed SGPLE magnitude and spatial distribution.

Averaged rank pseudo MRI showed significantly lower SGPLE compared with first rank pseudo MRI (Figures 1, 2). Moreover, lower ORE did not necessarily imply lower SGPLE (Figure 1). Therefore, ORE is not a sufficient indicative parameter for selecting the best pseudo MRI from the MRI dataset, i.e., first rank pseudo MRI. However, this was not the case for averaged rank pseudo MRI, which is not very dependent on ORE. SGPLE for averaged rank pseudo MRI exponentially decreased as the number of pseudo MRIs (**n**) ranked from 1 to 20 increased, and then stabilized afterward despite the further inclusion of higher rank pseudo MRIs that have a higher SGPLE (Figure 1). Taken together, these results support our hypothesis that source localization error for different pseudo MRIs is likely to be in different orientations. Therefore, the error is neutralized to a certain extent after the averaging of multiple pseudo MRIs, bringing about an approximate model (i.e., averaged rank pseudo MRI) that is closer to the real one.

The mean upper bound of approximate source localization error (i.e., SGPLE) at the individual level was about 10 mm for the averaged rank pseudo MRI (Figure 3), better than or close to that observed in previous studies (Van 't Ent et al., 2001; Holliday et al., 2003; Darvas et al., 2006; Valdés-Hernández et al., 2009). However, many of these studies focused on EEG applications whereas the present study is mainly focused on MEG applications. Compared to EEG, MEG-based source error is less conduction model sensitive; therefore, the inter-subject variability in corresponding source locations in the sourcemodel mainly contributes to source localization error. Moreover, EEG-based studies more often use the non-linear warping-based methods to generate pseudo MRI, taking into consideration the electrode positions over the scalp. In contrast to the rigid body affine transform with scaling, warping transformation also takes into consideration the head shape information during pseudo MRI approximation. However, this approach is more sensitive to a number of sampling points and spatial regularity on head surfaces. Moreover, the accuracy of warping transformation is more reliable at points closest to the scalp, and may lead to over-correction in deeper brain structure (Valdés-Hernández et al., 2009). In contrast, we used a larger number of MRIs (head shapes) with scaling, which effectively widens the search space and offers one solution to matching the subject's head shapes more closely. Thus, this excludes the necessity of warping and, hence, the problem of over- or under-correction without compromising head shape information. Moreover, the proposed principle and approach of averaged rank pseudo MRI can be extended for EEG applications, but needs to take various issues into consideration, particularly the assumptions of linearity during averaging of the headmodel to generate pseudo-realistic models (Guimond et al., 2000; Christensen et al., 2006; Valdés-Hernández et al., 2009).

MEG-based studies frequently involve multiple subjects and the conclusion is often drawn from a mean group effect. One of the advantages of the present study is that it provides some quantitative analysis of functional source localization error between real and pseudo MRIs for functional MEG data, which is the closest to practical scenarios. Moreover, the use of a visual stimulus that recruits primary visual cortex offers two advantages. First, it is less prone to inter-subject functional and anatomical variability (Hinds et al., 2009; Nieto-Castanon and Fedorenko, 2012); therefore, results are more representative of model approximation error. Second, the primary visual cortex showed higher model approximation error (i.e., SGPLE), thus reducing the bias toward getting lower peak source localization error. In the present study, we observed broadly similar functional group response distribution (at a latency of peak ERP activity) in the visual cortex in response to visual stimuli for real and pseudo MRIs. It suggests that the effect of functional inter-subject variability on group source response is sufficiently larger, in such a way that the source localization error arising from the use of pseudo MRI exerts a minimal effect on group functional response. However, the averaged rank pseudo MRI, compared with the first rank pseudo MRI, showed better performance in terms of peak source localization error (Figures 5A,B), which is in line with the observed source grid point location error (SGPLE, Figure 3) and centroid error (CE, Figure 4) performance. The interpretation of the difference in source strength and source overlap between real and pseudo MRIs is not straightforward, given the complex relationship between spatial characteristics of the source distribution, peak source activity, and strength of source activity. However, higher source strength (in the left visual cortex) along with lower peak localization error also endorses the averaged rank pseudo MRI over the first rank pseudo MRI (Figure 6).

There exists inter-subject functional variability, which is a limitation in trying to take complete advantage of real MRI because perfect registration of MRI and MEG sensors is not guaranteed, and limitation of spatial resolution of inverse methodology (i.e., MNE solution in the current study). Consequently, there was a subtle difference in functional group source activity between pseudo MRI and real MRI that is less likely to affect the general interpretation of group effects, at least for functional visual stimulation response as used in the present study. Also, averaged rank pseudo MRI has an advantage over first rank pseudo MRI in terms of model approximation error that is also reflected in functional peak source localization error. Taken together, these facts support the use of pseudo MRI, particularity averaged rank pseudo MRI, over real MRI for functional group MEG studies.

In the present work, the population from which digitized head anatomy and MRI dataset recorded were from the same ethnic group. Racial or ethnic differences between the target population (digitized head anatomy) and the population from which the MRI dataset was recorded may influence the source localization error (anatomical). However, Valdés-Hernández et al. (2009) reported a non-significant impact of race and gender on headmodel approximation. Therefore, in the case of non-availability of MRI dataset from an ethnic group similar to the target population, we may use another available MRI dataset (e.g., HCP MRI dataset or other MRI scans recorded from a population as ethnically close as possible to the target population), without much influencing the outcome. Moreover, it is not very difficult to create such an MRI database specific to a country or an ethnic group.

In conclusion, pseudo MRI, particularly the averaged rank pseudo MRI approximated from an available MRI dataset, can be a reliable substitute in the absence of real MRI or to bypass the necessity of real MRI for participants in group-based MEG studies. It can achieve this without considerably affecting the generality of the functional group source response.

## Ethics statement

We used online publicly available data recorded from human.

## Author contributions

BG conceived and designed the research, BG, SL, MK, HK, and KK performed the analysis, prepared the figures and wrote the manuscripts. All authors approved the final version of the manuscript.

### Conflict of interest statement

The authors declare that the research was conducted in the absence of any commercial or financial relationships that could be construed as a potential conflict of interest.

## References

[B1] Chiarelli A. M. Maclin E. L. Low K. A. Fabiani M. Gratton G. (2015). Comparison of procedures for co-registering scalp-recording locations to anatomical magnetic resonance images. J. Biomed. Opt. 20:16009 10.1117/1.JBO.20.1.016009 PMC4288136 25574993

[B2] Christensen G. E. Johnson H. J. Vannier M. W. (2006). Synthesizing average 3D anatomical shapes. Neuroimage 32, 146–158. 10.1016/j.neuroimage.2006.03.018 16697223

[B3] Darvas F. Ermer J. J. Mosher J. C. Leahy R. M. (2006). Generic head models for atlas-based EEG source analysis. Hum. Brain Mapp. 27, 129–143. 10.1002/hbm.20171 16037984 PMC6871464

[B4] Fuchs M. Kastner J. Wagner M. Hawes S. Ebersole J. S. (2002). A standardized boundary element method volume conductor model. Clin. Neurophysiol. 113, 702–712. 10.1016/S1388-2457(02)00030-5 11976050

[B5] Ghosh S. S. Kakunoori S. Augustinack J. Nieto-Castanon A. Kovelman I. Gaab N.. (2010). Evaluating the validity of volume-based and surface-based brain image registration for developmental cognitive neuroscience studies in children 4 to 11 years of age. Neuroimage 53, 85–93. 10.1016/j.neuroimage.2010.05.075 20621657 PMC2914629

[B6] Gross J. Baillet S. Barnes G. R. Henson R. N. Hillebrand A. Jensen O.. (2013). Good practice for conducting and reporting MEG research. Neuroimage 65, 349–363. 10.1016/j.neuroimage.2012.10.001 23046981 PMC3925794

[B7] Guimond A. Meunier J. Thirion J.-P. (2000). Average brain models: a convergence study. Comput. Vis. Image Underst. 77, 192–210. 10.1006/cviu.1999.0815

[B8] Hinds O. Polimeni J. R. Rajendran N. Balasubramanian M. Amunts K. Zilles K.. (2009). Locating the functional and anatomical boundaries of human primary visual cortex. Neuroimage 46, 915–922. 10.1016/j.neuroimage.2009.03.036 19328238 PMC2712139

[B9] Holliday I. E. Barnes G. R. Hillebrand A. Singh K. D. (2003). Accuracy and applications of group MEG studies using cortical source locations estimated from participants' scalp surfaces. Hum. Brain Mapp. 20, 142–147. 10.1002/hbm.10133 14601140 PMC6872117

[B10] Labadie R. F. Shah R. J. Harris S. S. Cetinkaya E. Haynes D. S. Fenlon M. R.. (2004). Submillimetric target-registration error using a novel, non-invasive fiducial system for image-guided otologic surgery. Comput. Aided Surg. 9, 145–153. 10.3109/10929080500066922 16192054

[B11] Larson-Prior L. J. Oostenveld R. Della Penna S. Michalareas G. Prior F. Babajani-Feremi A.. (2013). Adding dynamics to the human connectome project with MEG. Neuroimage 80, 190–201. 10.1016/j.neuroimage.2013.05.056 23702419 PMC3784249

[B12] Low K. (2004). Linear Least-Squares Optimization for Point-to-Plane ICP Surface Registration. Chapel Hill, NC: University of North Carolina Available online at: https://www.iscs.nus.edu.sg/~lowkl/publications/lowk_point-to-plane_icp_techrep.pdf

[B13] Nieto-Castanon A. Fedorenko E. (2012). Subject-specific functional localizers increase sensitivity and functional resolution of multi-subject analyses. Neuroimage 63, 1646–1669. 10.1016/j.neuroimage.2012.06.065 22784644 PMC3477490

[B14] Oostenveld R. Fries P. Maris E. Schoffelen J. M. (2011). FieldTrip: open source software for advanced analysis of MEG, EEG, and invasive electrophysiological data. Comput. Intell. Neurosci. 2011:156869 10.1155/2011/156869 PMC3021840 21253357

[B15] Park S. Y. Subbarao M. (2003). An accurate and fast point-to-plane registration technique. Pattern Recognit. Lett. 24, 2967–2976. 10.1016/S0167-8655(03)00157-0

[B16] Rusinkiewicz S. Levoy M. (2001). Efficient variants of the ICP algorithm, in Proceedings of International Conference on 3-D Digital Imaging and Modeling, 3DIM (Quebec), 145–152. 10.1109/IM.2001.924423

[B17] Tadel F. Baillet S. Mosher J. C. Pantazis D. Leahy R. M. (2011). Brainstorm: a user-friendly application for MEG/EEG analysis. Comput. Intell. Neurosci. 2011:879716 10.1155/2011/879716 PMC3090754 21584256

[B18] Valdés-Hernández P. A. von Ellenrieder N. Ojeda-Gonzalez A. Kochen S. Alemán-Gómez Y. Muravchik C.. (2009). Approximate average head models for EEG source imaging. J. Neurosci. Methods 185, 125–132. 10.1016/j.jneumeth.2009.09.005 19747944

[B19] Van Essen D. C. Smith S. M. Barch D. M. Behrens T. E. J. Yacoub E. Ugurbil K. (2013). The WU-Minn human connectome project: an overview. Neuroimage 80, 62–79. 10.1016/j.neuroimage.2013.05.041 23684880 PMC3724347

[B20] Van 't Ent D. De Munck J. C. Kaas A. L. (2001). A fast method to derive realistic BEM models for E/MEG source reconstruction. IEEE Trans. Biomed. Eng. 48, 1434–1443. 10.1109/10.966602 11759924

[B21] Wendel K. Väisänen O. Malmivuo J. Gencer N. G. Vanrumste B. Durka P. (2009). EEG/MEG source imaging: methods, challenges, and open issues. Comput. Intell. Neurosci. 2009:656092 10.1155/2009/656092 PMC2715569 19639045

[B22] Whalen C. Maclin E. L. Fabiani M. Gratton G. (2008). Validation of a method for coregistering scalp recording locations with 3D structural MR images. Hum. Brain Mapp. 29, 1288–1301. 10.1002/hbm.20465 17894391 PMC6871211

[B23] Xiong J. Rao S. Jerabek P. Zamarripa F. Woldorff M. Lancaster J.. (2000). Intersubject variability in cortical activations during a complex language task. Neuroimage 12, 326–339. 10.1006/nimg.2000.0621 10944415

