# Supplementary material for: Approximate Subject Specific Pseudo MRI from an Available MRI Dataset for MEG Source Imaging
Source: Front Neuroinform. 2017 Aug 8;11:50. doi: 10.3389/fninf.2017.00050 (PMC5550724; doi:10.3389/fninf.2017.00050)
Supplement: Supplementary file 1 [file Image1.PDF]

# Approximate subject specific pseudo MRI from available MRI dataset for MEG source imaging

**Figure 5. for 40 subjects in a group**

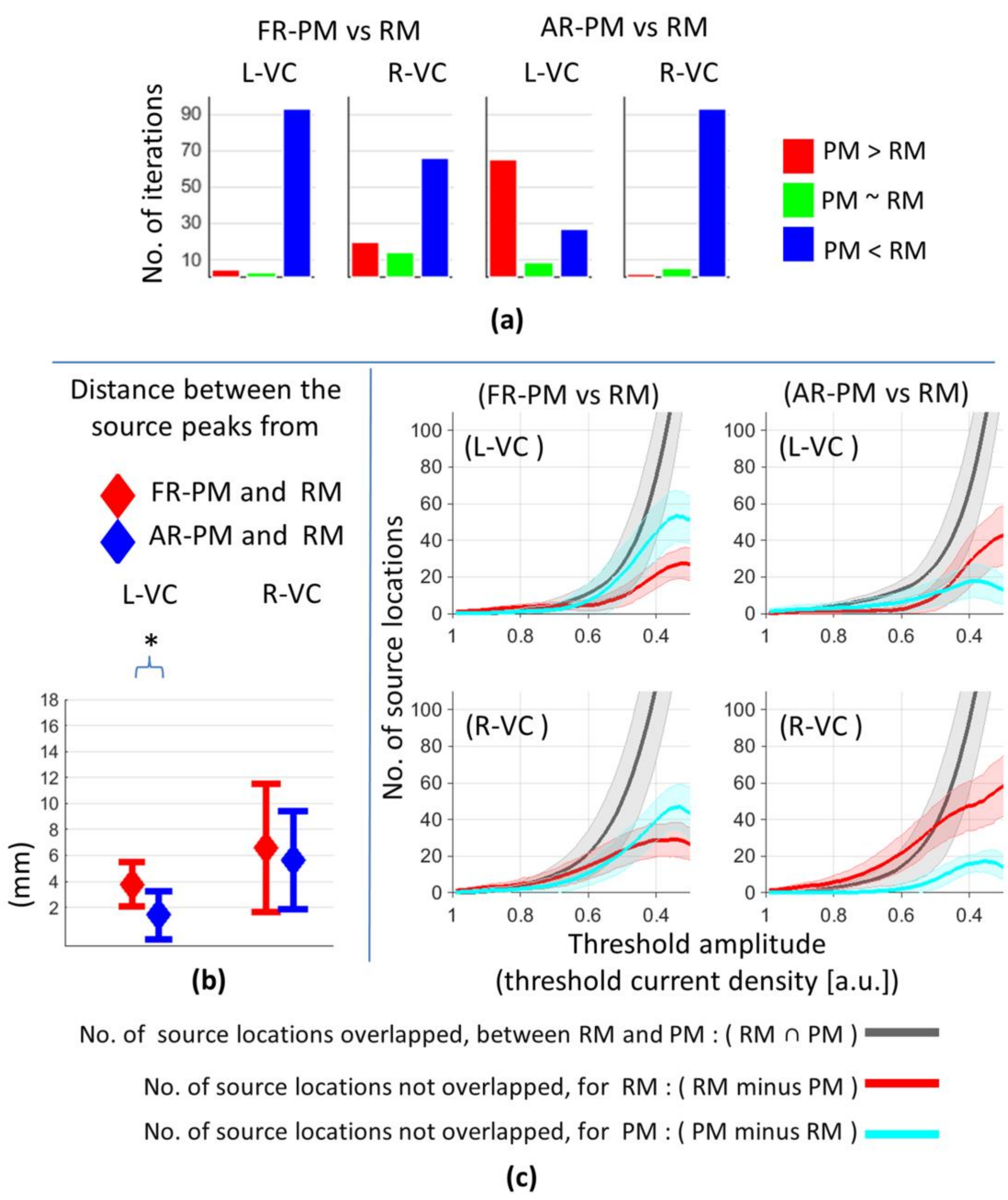


**Figure. S1.** Comparison of functional group source ERP response between pseudo MRI and real MRI for **40** subjects in a group and 100 group iterations. (a) Comparison of peak source ERP magnitude for different group iterations. Y-axis represents number of group iterations showed significant difference for peak source ERP magnitude ($p < 0.01$: blue and red bar, $p > 0.01$: green bar, *bootstrap approach*). (b) Peak source localization error across the group iterations (mean ± standard deviation). '*':- $p < 0.01$, *Wilcoxon signed-rank test*, $n = 100$ *group iterations*. (c) Overlap of activation region (no. of grid point locations) between real MRI and pseudo MRI after applying different levels of threshold value across the group iterations (mean ± standard deviation). RM:- real MRI, PM:- pseudo MRI, FR-PM:- first rank pseudo MRI, AR-PM:- averaged rank pseudo MRI, L-VC:- left visual cortex, R-VC:- right visual cortex, a.u.:- arbitrary unit where a.u.=1 corresponds to maximum source ERP value for a given group iteration in visual cortex for real MRI.

**Figure 5. for 60 subjects in a group**

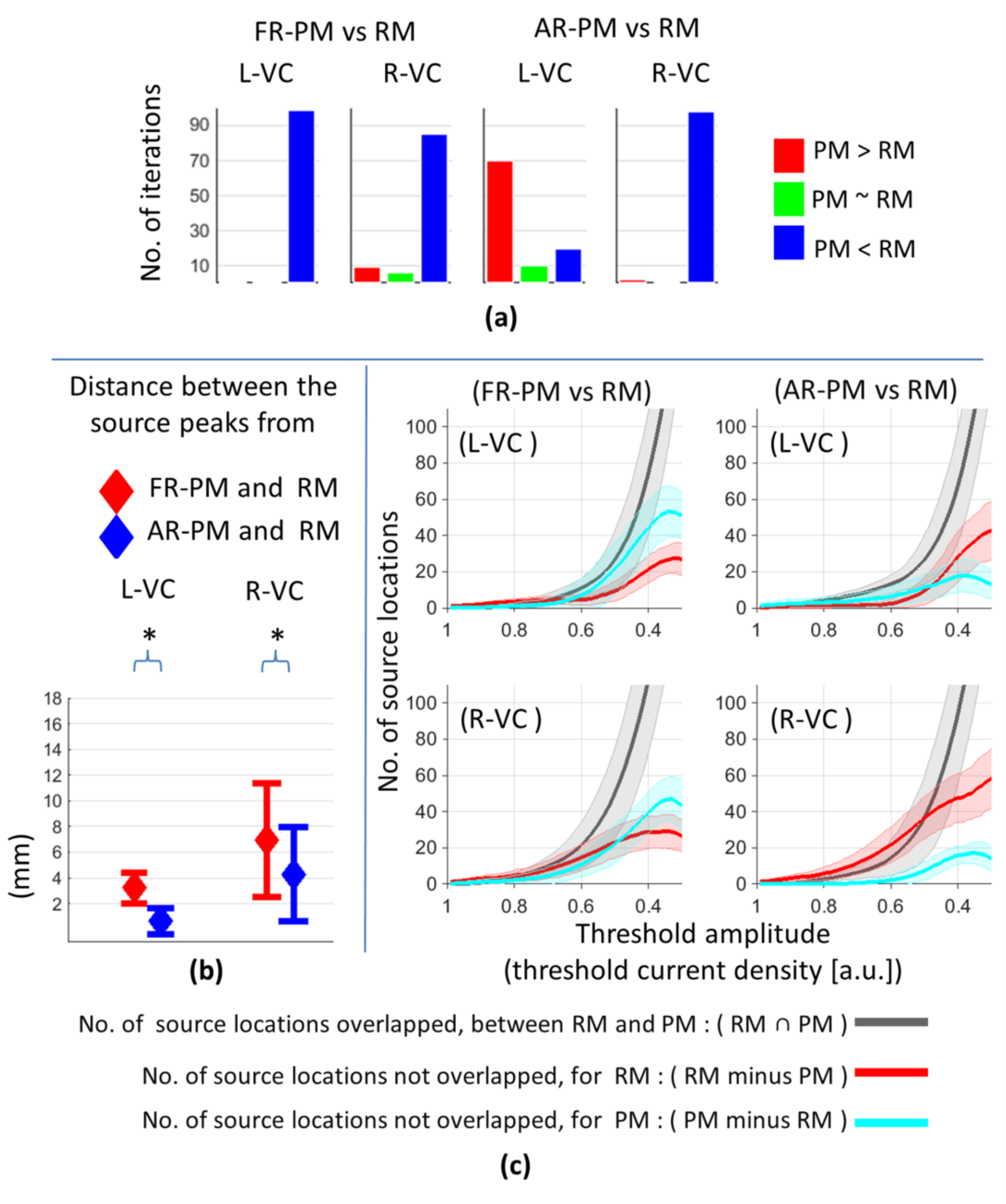


**Figure. S2.** Comparison of functional group source ERP response between pseudo MRI and real MRI for **60** subjects in a group and 100 group iterations. (a) Comparison of peak source ERP magnitude for different group iterations. Y-axis represents number of group iterations showed significant difference for peak source ERP magnitude ($p < 0.01$: blue and red bar, $p > 0.01$: green bar, *bootstrap approach*). (b) Peak source localization error across the group iterations (mean ± standard deviation). '*':- $p < 0.01$, *Wilcoxon signed-rank test*, $n = 100$ *group iterations*. (c) Overlap of activation region (no. of grid point locations) between real MRI and pseudo MRI after applying different levels of threshold value across the group iterations (mean ± standard deviation). RM:- real MRI, PM:- pseudo MRI, FR-PM:- first rank pseudo MRI, AR-PM:- averaged rank pseudo MRI, L-VC:- left visual cortex, R-VC:- right visual cortex, a.u.:- arbitrary unit where a.u.=1 corresponds to maximum source ERP value for a given group iteration in visual cortex for real MRI.
